# Supplementary material for: MetaRibo-Seq measures translation in microbiomes
Source: Nat Commun. 2020 Jun 29;11:3268. doi: 10.1038/s41467-020-17081-z (PMC7324362; doi:10.1038/s41467-020-17081-z)
Supplement: Supplementary file 10 — Supplementary Data 7 [file 41467_2020_17081_MOESM10_ESM.zip › File2/Confidence_High_Taxonomy/116240_out.krona.html]

Javascript must be enabled to view this page.

members
magnitude
magnitudeUnassigned
count
unassigned
taxon
rank

116240\_out

12

2

SRS018313\_contig\_number\_7303SRS893285\_contig\_number\_contig-100\_4077.4078

10
superkingdom
2

phylum
10
1239

class
10
186801

10
order
186802

8
family
541000

8
genus
216851

species
8
853

SRS020869\_contig\_number\_28149SRS054956\_contig\_number\_9362SRS063985\_contig\_number\_contig-100\_696.175300SRS143466\_contig\_number\_4465SRS144714\_contig\_number\_26715SRS147919\_contig\_number\_21021SRS148159\_contig\_number\_contig-100\_1409.339418SRS148319\_contig\_number\_16916

1
family
186803


SRS1055099\_contig\_number\_contig-100\_2373.92595
2292271
1
species

1
family
31979

genus
1
1485

59620
1
species

SRS149784\_contig\_number\_9866
